# Post Traumatic Growth: A Novel Framework to Strengthen Resilience and Heart Failure Self-Care

**DOI:** 10.1093/geroni/igaf122.3827

**Published:** 2025-12-31

**Authors:** Rebecca Meraz, Jocelyn McGee

**Affiliations:** Baylor University, Dallas, Texas, United States; Baylor University, Waco, Texas, United States

## Abstract

Despite the health benefits of heart failure (HF) self-care, more than 50% of patients are nonadherent to self-care practices. Many individuals struggle due to psychological distress, burden, and diminished resilience. Traditional interventions often neglect enhancing positive psychological growth as a strategy for improving self-care adherence. Using a case study methodology, we examined the experiences of two individuals participating in a resilience-based intervention to improve HF self-care to demonstrate the applicability of the PTG framework to future intervention design. The pilot intervention, StruggleStrong, was a structured 6-week, telehealth, program designed to bolster key aspects of resilience, including personal strengths, optimism, meaning in life, gratitude, and deeper relational connections. Our findings illustrated that participants’ experiences aligned seamlessly with the PTG framework. Growth in personal strength empowered participants to view HF self-care challenges as opportunities to apply their own capability. Leaning on faith and personal values helped participants transform the adversity of adhering to stressful self-care tasks into a deepened sense of meaning and purpose. Gratitude and appreciation in life were important to fostering positive psychological growth and resilience. Gratitude was also significant to relational growth, fostering a greater appreciation for the caregivers, doctors, and friends who were resources for navigating burdensome self-care tasks. A greater sense of confidence, purpose, and gratitude led to new possibilities and self-care goals. The PTG framework could be a novel framework for strengthening resilience and improving HF self-care.

